# The clinical characteristics and therapeutic outcomes of cryptococcal meningitis in elderly patients: a hospital-based study

**DOI:** 10.1186/s12877-019-1108-0

**Published:** 2019-03-25

**Authors:** Wan-Chen Tsai, Chia-Yi Lien, Jun-Jun Lee, Wen-Chiu Hsiao, Chi-Ren Huang, Nai-Wen Tsai, Chiung-Chih Chang, Cheng-Hsien Lu, Wen-Neng Chang

**Affiliations:** 1 Department of Neurology, Chang Gung Memorial Hospital-Kaohsiung, Chang Gung University College of Medicine, 123, Ta Pei Road, Niao Sung Hsiang, Kaohsiung, Taiwan; 2 Department of Information Management, National Sun Yat-sen University, Kaohsiung, Taiwan

**Keywords:** Cryptococcal meningitis, Elderly, Gender, Cryptococcemia, Cerebral infarction, Altered consciousness

## Abstract

**Background:**

The elderly, and especially those with an immuno-compromised status, are vulnerable to infectious diseases. The purpose of this study was to examine the clinical characteristics and therapeutic outcomes of cryptococcal meningitis (CM) in elderly patients in Taiwan.

**Methods:**

Ninety-nine adult patients with CM were identified during a 15-year study period (2002–2016), of whom 38 elderly (≥ 65 years) patients (16 men and 22 women, median age 72.9 years; range 65–86 years) were included for analysis. The clinical characteristics and therapeutic outcomes of these patients were analyzed and compared to non-elderly adult patients (< 65 years) with CM.

**Results:**

Among the 38 patients, diabetes mellitus was the most common underlying condition (15), followed by adrenal insufficiency (7), malignancy (6), hematologic disorders (5), chronic obstructive pulmonary disease (5), autoimmune diseases (3), liver cirrhosis (3) and acquired immunodeficiency syndrome (1). Altered consciousness (29), fever (21) and headache (17) were the leading clinical manifestations. Positive cerebrospinal fluid and blood cultures for *Cryptococcus* (*C*.) *neoformans* were found in 26 and 9 patients, respectively. There were significant differences in gender, altered consciousness and recent cerebral infarction between the elderly and non-elderly groups. The elderly group had a high mortality rate (36.8%, 14/38), and the presence of cryptococcemia was the most significant prognostic factor.

**Conclusions:**

This study offers a preliminary view of the clinical characteristics of CM in the elderly. The results suggest that elderly patients (≥ 65 years) are more vulnerable to CM than adults aged < 65 years. Compared to the non-elderly group, the elderly group had female predominance, higher rates of altered consciousness and recent cerebral infarction as the clinical presentation. The presence of cryptococcemia was a significant prognostic factor in the elderly group. This study is limited by the small number of patients, and further large-scale studies are needed to better delineate this specific infectious syndrome.

## Background

Aging is a complex process that negatively impacts the development of the immune system and its ability to function. In addition, concomitant disabilities and comorbidities are common in the elderly [1]. These factors render elderly individuals more vulnerable to infectious diseases including central nervous system (CNS) infections [1–5]. However, the classic presentations of infectious diseases are not always noted in elderly patients, making it difficult to make an early diagnosis leading to a delay in treatment [5–7]. Cryptococcal meningitis (CM), caused by *Cryptococcus neoformans* infection, is a serious infectious disease of the CNS [8] occurring in both immuno-compromised and immuno-competent patients [8, 9]. Clinically, in contrast to acute bacterial meningitis, patients with CM, which is classified as chronic meningitis, have a slower onset of symptoms, evolving over days to a few weeks [10]. Nevertheless, laboratory studies such as the measurement of cryptococcal antigen (Ag) titer and/or culture of *C. neoformans* are still the mainstay for a definite diagnosis. In our previous study of the clinical characteristics of bacterial meningitis in elderly patients [5], the clinical presentations were similar to those of non-elderly adults. To date, the clinical characteristics and therapeutic outcomes of CM in elderly patients have not been reported in the literature. Because of the aging population in Taiwan as well as in many other countries [5], we conducted this study to analyze the clinical and laboratory features and therapeutic outcomes of elderly patients with CM compared to non-elderly adults with CM in order to delineate the clinical characteristics of this specific group of patients.

## Methods

We retrospectively reviewed the clinical manifestations, laboratory data and initial neuroimaging features of adult patients (≥18 years of age) with a new diagnosis of CM admitted to Kaohsiung Chang Gung Memorial Hospital during a 15-year study period (2002–2016). A total of 99 patients were identified and classified into elderly (≥ 65 years) and non-elderly (< 65 years) groups. The therapeutic results of these patients were evaluated at discharge. This study was approved by the Ethics Committee of Kaohsiung Chang Gung Memorial Hospital (IRB No:1608300002).

In this study, CM was defined as either (1) isolation of *C. neoformans* in one or more cerebrospinal fluid (CSF) cultures, a positive CSF cryptococcal Ag titer, or positive CSF India ink staining which stains the polysaccharide capsule of *C. neoformans* and exhibits a halo around the cell against a black background, and clinical features of meningitis; or (2) isolation of *C. neoformans* in a blood culture with clinical presentations of meningitis and typical CSF features [8, 11]. The neuroimaging findings used for analysis were derived from initial cranial magnetic resonance imaging and/or cranial computed tomography studies as previously described [8]. During the study period, the main antifungal regimen was amphotericin B +/− flucytosine +/− fluconazole for induction, consolidation and maintenance therapy [8, 12]. Extraventricular drainage and/or ventriculoperitoneal shunts were used to relieve hydrocephalus and/or increased intracranial pressure [8, 12].

Because a cryptococcal Ag titer ≥1:1024 is an important cut-off point for the prognosis of CM [11], we used this value for further analytic comparisons. In this study, we performed two separate statistical analyses. Because immune condition is an important factor in the underlying condition of CM patients, we also classified the 99 enrolled adult CM patients into those with and without an immune-compromised state [13] for comparison. First, we compared the clinical characteristics, laboratory data and neuroimaging findings between the elderly and non-elderly groups. Second, we investigated the potential prognostic factors of the elderly group. In statistical analysis, categorical variables were analyzed using the chi-square test or Fisher’s exact test, and continuous variables were analyzed using the t-test. Variables with a *p*-value < 0.01 were further analyzed using multivariate logistic regression analysis.

## Results

**Fig. 1:**
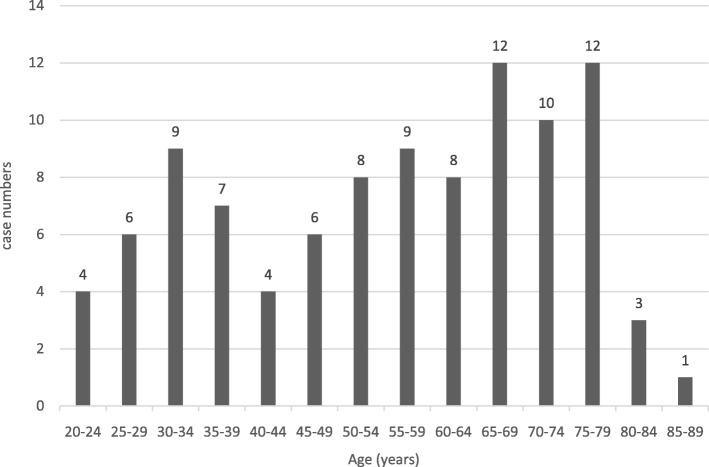
The age distribution of the 99 elderly patients with cryptococcal meningitis

Of the 99 adult patients with CM, 64 were men and 35 were women (age range 20–86 years; Fig. 1).Thirty-eight patients were classified as being elderly (≥65 years) and 61 as non-elderly (< 65 years). The 38 elderly patients included 16 men and 22 women (median age 72.9 years, range 65–86 years). Table 1 shows the comparisons of clinical characteristics, laboratory findings and neuroimaging features between the elderly and non-elderly groups. Of the underlying conditions, diabetes mellitus was the most common (23), followed by acquired immunodeficiency syndrome (AIDS) (12), hematologic disorders (12), autoimmune disorders (10), liver cirrhosis (9), malignancy (9), adrenal insufficiency (8) and chronic obstructive pulmonary disease (5). With regards to the clinical presentations, headache was the most common (65), followed by fever (60), altered consciousness (49), visual disturbance (16), seizures (15) and hearing impairment (6).

**Table 1:** The clinical characteristics and laboratory data of the 99 adults with cryptococcal meningitis

	Non-elderly group (*n* = 61)	Elderly group (*n* = 38)	*p*-value
Gender			
Male	48	16	< 0.001
Female	13	22	
Mortality at discharge	19	14	0.597
Underlying disease			
AIDS	11	1	0.026
Diabetes mellitus	8	15	0.003
Liver cirrhosis	6	3	1.000
Hematologic disorders	7	5	1.000
Autoimmune disorders	7	3	0.737
Malignancy	3	6	0.083
Chronic obstructive pulmonary disease	0	5	0.007
Adrenal insufficiency	1	7	0.005
Organ transplantation	1	0	1.000
Immunocompromised state	26	17	0.837
Initial presentation			
Headache	48	17	0.001
Altered consciousness	20	29	< 0.001
Fever	39	21	0.390
Seizure	9	6	0.889
Visual disturbance	15	1	0.004
Hearing impairment	5	1	0.402
Cerebrospinal fluid data			
White blood cell count (10^9^/L)	0.17	0.09	0.022
Glucose (mmol/L)	2.18	2.92	0.153
Protein (g/L)	1.50	2.54	0.186
Lactate (mmol/L)	5.25	5.35	0.896
Positive India ink stain	22	9	0.113
Cryptococcal Ag titer (≥1:1024)	26	12	0.163
Positive culture result	51	26	0.004
Serum cryptococcal Ag titer (≥1:1024)	22	12	0.766
Cryptococcemia	21	9	0.258
Extraneural involvement	5	5	0.501
Neuroimaging findings			
Basal meningeal enhancement	22	12	0.584
Pseudocyst/VR space dilatation	18	14	0.394
Hydrocephalus	18	15	0.263
Recent cerebral infarction	3	11	0.001
Cryptococcoma	5	1	0.404

*AIDS* acquired immunodeficiency syndrome, *Ag* antigen, *VR* Virchow-Robin

**Fig. 2:**
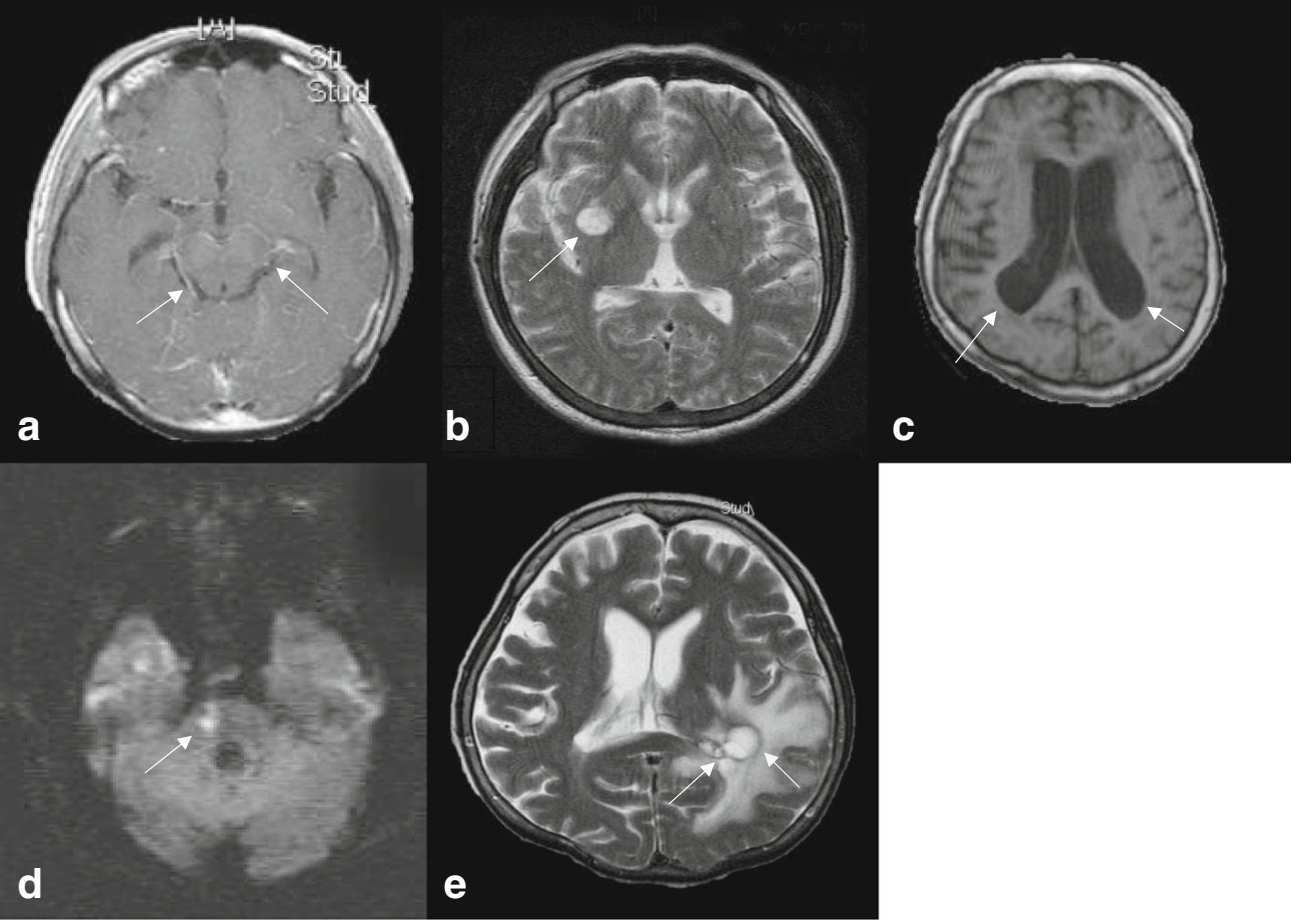
The main magnetic resonance imaging features of the elderly patients with cryptococcal meningitis. **a**: Gadolinium contrast-enhanced T1-weighted image (T1WI) showing basal meningeal enhancement (arrow). **b**: T2-weighted magnetic resonance image (T2WI) showing pseudocysts (arrow). **c**: T1WI showing ventricular dilatation. **d**: Diffusion-weighted image showing a recent cerebral infarct at the right pons (arrow). **e**: T2WI showing cryptococcoma (arrow)

With regards to the CSF data, the white blood cell count ranged from 0 to 0.85 × 10^9^/L (mean 0.14 × 10^9^/L), the glucose level ranged from 0.11 to 14.15 mmol/L (mean 2.38 mmol/L), the total protein level ranged from 0.06 to 26.26 g/L (mean 1.91 g/L), and the lactate level ranged from 1.44 to 28.97 mmol/L (mean 5.29 mmol/L). Thirty-eight of the 99 patients had a CSF cryptococcal Ag titer ≥1:1024, and a positive India ink stain was found in 31 patients. A serum cryptococcal Ag titer ≥1:1024 was found in 34 patients. Positive CSF and blood cultures of *C. neoformans* were found in 77 and 30 of the 99 patients, respectively. The main neuroimaging features (Fig. 2) of the 99 patients included basal meningeal enhancement (34), hydrocephalus (33), pseudocyst/Virchow-Robin space dilatation (32), recent cerebral infarction (14), and cryptococcoma (6).There were significant differences in gender, diabetes mellitus, chronic obstructive pulmonary disease, adrenal insufficiency, headache, altered consciousness, visual disturbance, positive CSF culture result, and the presence of recent cerebral infarction between the elderly and non-elderly groups (Table 1). After multivariate logistic regression analysis, gender (*p* = 0.004; 95% CI = 0.019–0.479; OR = 0.095 for male gender), altered consciousness (*p* = 0.010; 95% CI = 1.621–38.161; OR = 7.865) and recent cerebral infarction (*p* = 0.012; 95% CI = 2.162–472.854; OR = 31.973) remained significant factors. Despite treatment, 14 of the 38 elderly patients died. Table 2 shows comparisons of the prognostic factors between the survivors (*n* = 24) and non-survivors (*n* = 14). There was only a significant difference in the presence of cryptococcemia between these two groups.

**Table 2:** The prognostic factors of the 38 elderly patients with cryptococcal meningitis

	Survivors (*n* = 24)	Non-survivors (*n* = 14)	*p*-value
Gender			
Male	11	5	
Female	13	9	0.542
Underlying disease			
AIDS	0	1	0.368
Diabetes mellitus	9	6	0.744
Liver cirrhosis	1	2	0.542
Hematologic disorders	4	1	0.633
Autoimmune disorders	1	2	0.542
Malignancy	3	3	0.650
Chronic obstructive pulmonary disease	1	4	0.052
Adrenal insufficiency	3	4	0.387
Immunocompromised state	7	10	0.011
Initial presentation			
Headache	11	6	0.859
Altered consciousness	17	12	0.438
Fever	11	10	0.126
Seizure	4	2	1.000
Visual disturbance	1	0	1.000
Hearing impairment	0	1	0.368
Cerebrospinal fluid data			
White blood cell count (10^9^/L)	0.09	0.09	0.950
Glucose (mmol/L)	2.77	3.21	0.664
Protein (g/L)	2.65	2.32	0.835
Lactate (mmol/L)	5.36	5.34	0.982
Positive India ink stain	3	6	0.050
Cryptococcal Ag titer (≥1024)	5	7	0.071
Positive culture result	16	10	0.711
Serum cryptococcal antigen titer (≥1024)	5	7	0.130
Cryptococcemia	2	7	0.006
Extraneural involvement	3	2	1.000
Image finding			
Basal meningeal enhancement	8	4	1.000
Pseudocyst/VR space dilatation	10	4	0.365
Hydrocephalus	9	6	0.823
Recent cerebral infarction	5	6	0.268
Cryptococcoma	1	0	1.000

*AIDS* acquired immunodeficiency syndrome, *Ag* antigen, *VR* Virchow-Robin

## Discussion

In 2018, more than 14% of the 23 million people in Taiwan were ≥ 65 years of age, and more people were aged ≥65 years than < 15 years, meaning that Taiwan has become an aged society [14]. In this study, 38.4% (38/99) of the adult patients with CM were elderly (≥ 65 years), indicating that this age group were more vulnerable to CM. This epidemiologic significance has not been reported in other Asian countries with an aged society or Western countries such as the United States [15–19].

In the current study, headache, fever and altered consciousness were the main clinical presentations of the 38 elderly patients with CM. These clinical presentations were similar to those of the non-elderly adults with CM (Table 1), and to both elderly and non-elderly adults with acute bacterial meningitis [5, 20]. Therefore, it is difficult to differentiate the exact type of meningitis if only the clinical presentations are considered. To avoid a missed or delayed diagnosis of CM in the elderly, keeping this specific infectious syndrome in mind and conducting appropriate studies for CNS infections are needed, especially in elderly patients with altered consciousness and/or headache and/or fever.

In this study, gender, altered consciousness and recent cerebral infarction were significantly different between the elderly and non-elderly groups. Although the sample size was not large and few variables were considered for the multivariate logistic regression analysis, based on the stepwise procedures, only three variables were selected as important predictive variables. Therefore, the maximum likelihood estimates of the coefficients were valid in the analysis.

In Taiwan, the retirement age is 65 years [21]. Previous studies have reported that more males are affected by cryptococcosis than females, and this disparity is seen in both HIV-positive and HIV-negative patients with cryptococcal infections [22]. We also reported the same male predominance in CM patients in our previous study [8], and a study from China also reported similar findings [15]. However, in the present study, there was a significant difference in gender between the elderly and non-elderly groups, with more females in the elderly group (57.9%, 22/38) and more males in the non-elderly group (78.7%, 48/61). Although a sex difference in the genetic architecture of susceptibility to *C. neoformans* infection was reported in an animal study [23], this difference has not been reported in clinical studies of elderly patients with CM. Altered consciousness as the initial presentation in CM is known to be an important prognostic factor for this serious infectious disease [11]. In the current study, significantly more of the elderly group had altered consciousness as the initial presentation compared to the non-elderly group (*p* < 0.001) and the patients with CM overall in our previous study [8]. Cerebral infarction is an important but frequently ignored finding in CM [8, 24], and it is also a significant prognostic factor of this serious infectious disease. In this study, the presence of cerebral infarction was a significant clinical feature in the elderly group. With the increasing size of the elderly population and evolving neuroimaging technology, silent cerebral infarction has garnered a lot of attention [25].The prevalence of cerebral infarction in the elderly population is known to increase steadily with age, and well-known cardiovascular risk factors and the metabolic syndrome are also important risk factors for the development of cerebral infarction in the elderly [25]. Our previous study revealed that old age was a significant factor for the development of cerebral infarction in CM patients [26]. In addition to age, many other factors may also play a role in the disturbance of cerebral hemodynamics and the subsequent development of cerebral infarction in patients with CM [24, 26], in which obvious basal meningeal enhancement as shown in brain MRI may play a significant role [8, 24, 26]. Increased basal meningeal enhancement is an important finding in brain MRI and confirms that inflammatory reactions are most intense in the basal meninges of patients with CM [8, 24, 26], and that local lenticulostriate and thalamoperforating arterioles are affected by such inflammatory processes with the subsequent development of cerebral infarction.

Among the 38 elderly patients with CM, 14 died during the therapeutic course, for a high mortality rate of 36.8%. As shown in Table 2, the presence of cryptococcemia was a significant prognostic factor for this specific group of patients, and it was present in 50% (7/14) of the non-survivors and 8.3% (2/24) of the survivors. The presence of cryptococcemia is a serious infectious condition, and patients with cryptococcemia have been reported to have a grave prognosis [27–29]. Therefore, CM patients with concomitant *C. neoformans* bloodstream infections should receive more aggressive and adequate treatment [28, 29].

## Conclusions

This preliminary study of CM in elderly patients revealed that 38.4% of the adult patients with CM were elderly, suggesting that the elderly are more vulnerable to the development of CM, at least in Taiwan. There were significantly more female patients in the elderly group with CM, and more male patients in the non-elderly group with CM. Beside the gender difference, the elderly group had significantly higher incidence rates of altered consciousness and cerebral infarction as the clinical presentations. The mortality rate of the elderly patients with CM was high in this study, and the presence of cryptococcemia was an important prognostic factor for this specific group of patients. This study is limited by the small number of patients, and further large-scale studies are needed to better delineate this specific infectious syndrome.

## Abbreviations

CM: Cryptococcal meningitis
CSF: Cerebrospinal fluid
